# Measurement of the Axial Magnetic Susceptibility of m-SWCNTs at High Temperatures in a Magnetic Field-Assisted FC-CVD

**DOI:** 10.3390/ma17112745

**Published:** 2024-06-04

**Authors:** Tanze Shen, Qiang Fu, Chunxu Pan

**Affiliations:** School of Physics and Technology, Wuhan University, Wuhan 430072, China; 17839175267@163.com (T.S.); fuqiang@whu.edu.cn (Q.F.)

**Keywords:** metallic single-walled carbon nanotubes, m-SWCNTs flow, trajectory, magnetic susceptibility

## Abstract

We synthesized some SWCNTs films under different magnetic fields and temperatures in a magnetic field-assisted FC-CVD and obtained Raman spectra of the films. By analyzing the Raman spectra, it was concluded that the SWCNTs films had defects, and the relative content of m-SWCNTs in the SWCNTs films was obtained. The trajectory of m-SWCNTs was obtained by analyzing the motion behavior of m-SWCNTs flow in the field-assisted system, and a model was built to describe the relationship between the relative content of m-SWCNTs and magnetic fields. The axial magnetic susceptibility of m-SWCNTs as a parameter was obtained by fitting the experimental results and the model. This is the first time that the axial magnetic susceptibility of m-SWCNTs has been obtained. The result obtained at 1273 K is at least two orders of magnitude greater than the magnetic susceptibilities and anisotropies of purified m-SWCNTs at 300 K, indicating that the defects increase the Curie temperature and Curie constant of m-SWCNTs. This is consistent with the spin-polarized density functional theory, which predicts that m-SWCNTs with vacancies have local magnetic moments around the vacancies and exhibit ferro- or ferrimagnetism.

## 1. Introduction

Since Iijima’s discovery of carbon nanotubes more than 30 years ago [1], researchers have carried out a lot of work in this area. Carbon nanotubes (CNTs) have attracted much attention due to their unique structures and properties. In general, CNTs are divided into two categories, i.e., multi-walled carbon nanotubes (MWCNTs) and single-walled carbon nanotubes (SWCNTs). According to their spatial structure and band structure, SWCNTs can be divided into two subspecies: metallic SWCNTs (m-SWCNTs) and semiconducting SWCNTs (s-SWCNTs) [2], which are represented by a chiral index (m, n). For m-SWCNTs, m − n = 3k, and for s-SWCNTs, m − n = 3k ± 1. The magnetic properties of CNTs have attracted much attention. However, there are few experiments on the magnetic susceptibilities of SWCNTs.

In theory, Lu [3] used the tight-binding model and London approximation to calculate the magnetic properties of SWCNTs. Ajiki et al. [4,5] used the *k*·*p* perturbation method to calculate their magnetic susceptibility. They both found that m-SWCNTs and s-SWCNTs showed different magnetic properties. On the one hand, m-SWCNTs exhibit a positive axial magnetic susceptibility ($\chi_{∥}$, for the magnetic field parallel to the tube axis) and a negative radial magnetic susceptibility ($\chi_{⊥}$, for the magnetic field perpendicular to the tube axis). On the other hand, the $\chi_{∥}$ and $\chi_{⊥}$ of s-SWCNTs are negative. The magnetic susceptibility of SWCNTs is proportional to the diameter of them ($d_{t}$). Marques et al. [6] calculated the magnetic susceptibilities of some zigzag s-SWCNTs by *ab* initio calculations and found that the magnetic susceptibility varies slightly depending on the value of m-n. Ma et al. [7] concluded that m-SWCNTs with vacancies exhibit ferro- or ferrimagnetism using the spin-polarized density functional theory.

In the experiment, the magnetic measurement of SWCNTs was divided into spectral analysis [8,9,10,11] and magnetization curve measurement [12,13,14,15]. Zaric et al. [8] estimated the magnetic susceptibility anisotropy $\left(∆\chi =\chi_{∥}-\chi_{⊥}\right)$ of SWCNTs using magnetophotoluminescence. Islam et al. [9] found that a fraction of acid-purified SWCNTs contain both linear-orbital and ferromagnetic anisotropies. Torrens et al. [10] measured $∆\chi /d_{t}$ using species-resolved polarized PL measurements. Searles et al. [11] extracted the ∆χ of m-SWCNTs through magnetic linear dichroism spectroscopy. Kim et al. [12] obtained a diamagnetic response from SWCNTs purified by air oxidation and chemical treatment with magnetic gradient filtration. Zaka et al. [14] reported that metal impurities in SWCNTs cause the observable Electron Paramagnetic Resonance signal. Nakai et al. [15] obtained the magnetic susceptibilities of SWCNTs purified by acid treatment and density gradient ultracentrifugation (DGU) without distinguishing $\chi_{∥}$ and $\chi_{⊥}$. The measurement of the magnetic susceptibility of SWCNTs is influenced by the residual catalyst particles. By analyzing the spectrum, the order parameters of SWCNTs were obtained, and the ∆χ of SWCNTs were derived from order parameters. By measuring the magnetization curve of SWCNTs, the magnetic susceptibilities of SWCNTs were obtained without distinguishing $\chi_{∥}$ and $\chi_{⊥}$. The results were linear combinations of $\chi_{∥}$ and $\chi_{⊥}$ from both spectral analysis and magnetization curve measurement. There is a lack of measurement of the axial magnetic susceptibility of SWCNTs at present.

In this paper, we fabricated SWCNTs films with different m-SWCNTs content using a self-made magnetic field-assisted FC-CVD and measured the $\chi_{∥}$ of m-SWCNTs by analyzing the motion behavior of m-SWCNTs flow in the field-assisted system. In our previous work, s-SWCNTs with 99% purity were fabricated in this device [16]. For FC-CVD, the carbon-containing organic matter (ethanol, methane, thiofuran, etc.) as the raw material and the catalyst precursor (ferrocene, nickelocene, etc.) flowed into the high-temperature reaction area along with the carrier gas. In the high-temperature reaction area, the raw materials decomposed into carbon atoms, and the catalyst particles were generated from the catalyst precursor, respectively. The carbon atoms were deposited on the catalyst particles, and SWCNTs were synthesized. The SWCNTs flowed into the film-forming area along with the carrier gas and were deposited on the substrate to form the films. Thermal motion was the driving force of the film-forming procession in the FC-CVD. For the field-assisted FC-CVD, a magnetic field was added to the film-forming area, and the magnetic force was the major driving force of the film-forming procession. We analyzed the motion behavior of m-SWCNTs, associated it with m-SWCNTs content, and obtained the $\chi_{∥}$ of m-SWCNTs at high temperatures.

## 2. Materials and Methods

**Figure 1:**
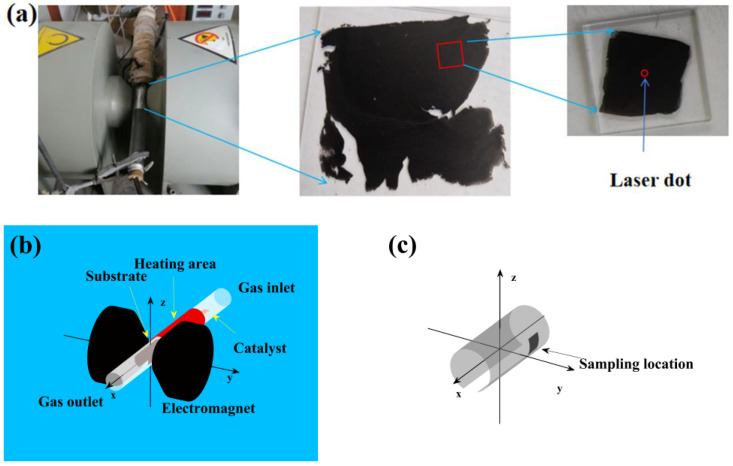
(**a**) Experimental device and sampling process. (**b**) Schematic diagram of experimental device. (**c**) Schematic diagram of sampling location.

As shown in Figure 1a, SWCNTs films were prepared by using a self-made magnetic field-assisted FC-CVD system. A magnetic field that was continuously adjusted from 0 T to 1.0 T was added to the film-forming area. The electromagnet (PEM-60, Litian Magnetoelectrican Science & Technology Co., Ltd., Mianyang, China) with a magnetic gap of 3.6 cm provided the magnetic field. The experimental conditions were as follows: ethanol (Sinopharm Tech Holdings Limited, Wuhan, Hubei, China) was used as the raw material, a mixture of ferrocene (Meryer Chemical Technology Co., Ltd., Shanghai, China) and sulfur powder (Alfa aesar Chemical Co., Ltd., Shanghai, China) with mass ratio of 1:1 was used as the catalyst precursor, 6.0 × 7.0 cm aluminum foil was used as the substrate, the inner radius of the glass tube was 1.25 cm, and a mixture of Ar and H_2_ with volume ratio of 5:1 was used as the carrier with a flow rate of 750 mL/min. SWCNTs were synthesized at different temperatures (1223 K, 1273 K, and 1323 K), and SWCNTs films were formed in different magnetic fields (central magnetic induction intensity: $B_{c}=0\;T,\;0.2\;T,\;0.4\;T,\;0.6\;T,\;0.8\;T,\;\mathrm{and}\;1.0\;T$). Then, the films were heated to 573 K for 2 h of holding and cooled to room temperature naturally. As shown in Figure 1, we took some 1.0 × 1.0 cm films from the position (x=−1.5 cm, y=1.25 cm, and z=0 cm) on the heat-treated films and placed them in a dilute hydrochloric acid solution for 4 h to dissolve the aluminum foils. Then, 1.5 × 1.5 cm quartz pieces were used to extract the films from the dilute hydrochloric acid.

The morphologies of the samples (see Appendix A) were characterized using a scanning electron microscope (SEM) (S-4800, Hitachi, Tokyo, Japan). The Raman spectra of the samples were obtained under a He-Ne laser (LabRAM HR Evolution, Hubei Nuclear Solid Physics Key Laboratory at Wuhan University, Wuhan, China) at 633 nm excitation through a 100× objective with a power of 1.9 mW.

## 3. Results

### 3.1. Characterization

**Figure 2:**
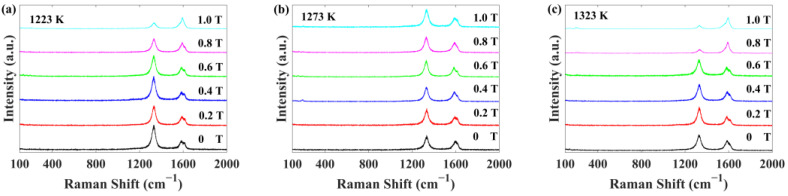
Raman spectra of SWCNTs films synthesized under different temperatures and magnetic fields: (**a**) 1223 K, (**b**) 1273 K, and (**c**) 1323 K.

Figure 2 illustrates the Raman spectra of the SWCNTs films. The characteristic peaks of SWCNTs generally fall into two categories. Radial breathing mode (RBM) signals appear in the range of 100 cm^−1^ to 350 cm^−1^, and their frequencies $\omega_{RBM}$ are inversely proportional to the diameter $d_{t}$ of the SWCNTs ($\omega_{RBM}=248/d_{t}\;{\mathrm{cm}}^{-1}$) [17]. For the Raman spectra with a laser wavelength of 633 nm, the RBM peaks of s-SWCNTs are in the range of 120–180 cm^−1^, and the RBM peaks of m-SWCNTs are in the range of 180–240 cm^−1^ [18]. High-energy mode (HEM) signals occur in the range of 1500 cm^−1^ to 1650 cm^−1^, known as G peaks. The G peaks of SWCNTs usually split into two peaks, i.e., the low frequency as $G^{-}$ peaks and the high frequency as $G^{+}$ peaks. The D peaks ($\omega_{D}=1300\;{\mathrm{cm}}^{-1}$) are related to the defects. The SWCNTs films, synthesized at 1 T and 1223 K, 0.8 T and 1323 K, and 1.0 T and 1323 K, have fewer defects.

**Figure 3:**
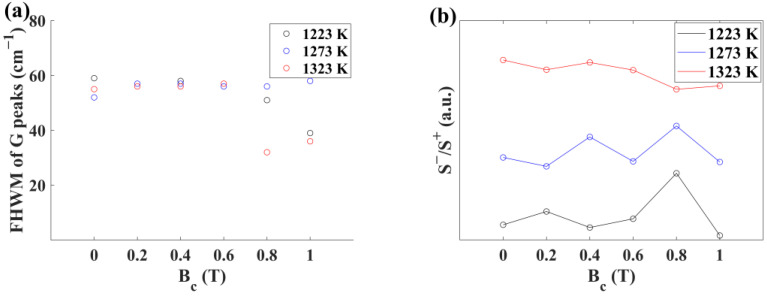
(**a**) FHWM of G peaks of SWCNTs films synthesized under different temperatures and magnetic fields. (**b**) The curves of $S^{-}/S^{+}$ varying with the magnetic field.

**Figure A2:**
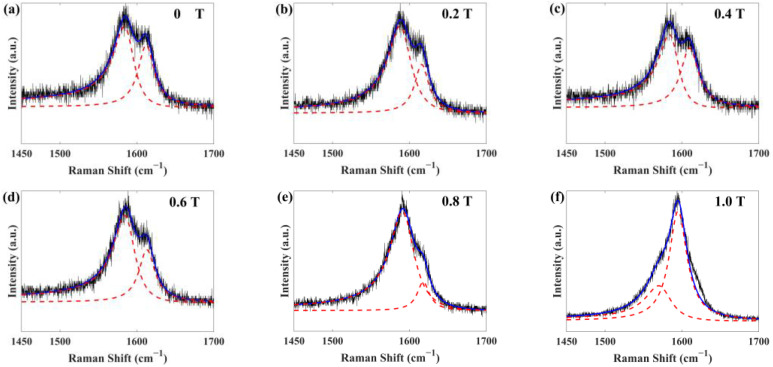
G peaks of SWCNTs films synthesized at 1223 K under different magnetic fields. (**a**) 0 T, (**b**) 0.2 T, (**c**) 0.4 T, (**d**) 0.6 T, (**e**) 0.8 T, (**f**) 1.0 T. The black lines are Raman spectra of G peaks. The blue lines are the fitting curves of G peaks. The red lines are the fitting curves of $G^{+}$ and $G^{-}$ peaks.

**Figure A3:**
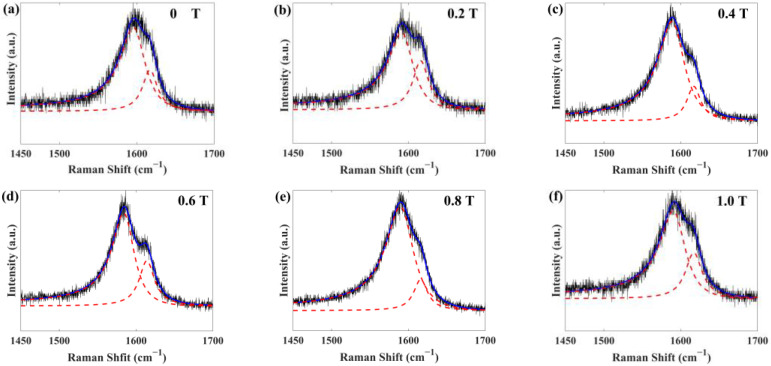
G peaks of SWCNTs films synthesized at 1273 K under different magnetic fields (**a**) 0 T, (**b**) 0.2 T, (**c**) 0.4 T, (**d**) 0.6 T, (**e**) 0.8 T, (**f**) 1.0 T. The black lines are Raman spectra of G peaks. The blue lines are the fitting curves of G peaks. The red lines are the fitting curves of $G^{+}$ and $G^{-}$ peaks.

**Figure A4:**
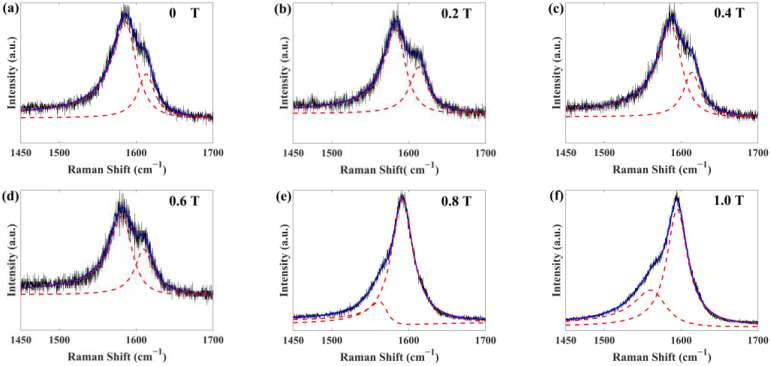
G peaks of SWCNTs films synthesized at 1323 K under different magnetic fields. (**a**) 0 T, (**b**) 0.2 T, (**c**) 0.4 T, (**d**) 0.6 T, (**e**) 0.8 T, (**f**) 1.0 T. The black lines are Raman spectra of G peaks. The blue lines are the fitting curves of G peaks. The red lines are the fitting curves of $G^{+}$ and $G^{-}$ peaks.

The G peaks of s-SWCNTs, corresponding to $G^{+}$ peaks, are Lorentz lines, and the G peaks of m-SWCNTs, corresponding to $G^{-}$ peaks, are BWF (Breit–Wigner–Fano) lines [19]. The fitting formula is as follows:

(1) $$I\left(\omega \right)=I^{+}\frac{1}{1+{\left[2\left(\omega -\omega^{+}\right)/\Gamma^{+}\right]}^{2}}+I^{-}\frac{{\left[1+2\left(\omega -\omega^{-}\right)/q\Gamma^{-}\right]}^{2}}{1+{\left[2\left(\omega -\omega^{-}\right)/\Gamma^{-}\right]}^{2}}$$

where $I^{+}$ is the intensity of the $G^{+}$ peak, $\omega^{+}$ is the $G^{+}$ peak frequency at the maximum intensity, $\Gamma^{+}$ is the full width at half maximum (FWHM) of the $G^{+}$ peak, $I^{-}$ is the intensity of the $G^{-}$ peak, $\omega^{-}$ is the $G^{-}$ peak frequency at the maximum intensity, $\Gamma^{-}$ is the FHWM of the $G^{-}$ peak, and $q^{-1}$ is the measure of the interaction of the phonon with a continuum of states. As shown in Figure A2, Figure A3 and Figure A4 (see Figure A2, Figure A3 and Figure A4 in Appendix B), we fitted the G peaks with Formula (1). The fitting data are shown in Table 1. $S^{+}$ is the area of the $G^{+}$ peaks, and $S^{-}$ is the area of the $G^{-}$ peaks. Figure 3a illustrates the FHWM of the G peak. The FHWM of the G peaks widened as the defects increased. Figure 3b illustrates $S^{-}/S^{+}$ varying with the magnetic field. The $S^{-}/S^{+}$ of SWCNTs films synthesized at 1223 K has a peak at 0.8 T. The $S^{-}/S^{+}$ of SWCNTs films synthesized at 1273 K has two peaks at 0.4 T and 0.8 T. The $S^{-}/S^{+}$ of SWCNTs films synthesized at 1323 K has no peak.

**Figure 4:**
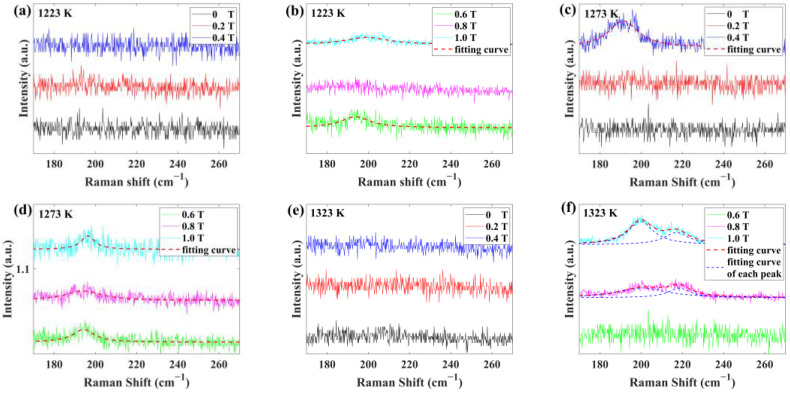
RBM peaks of SWCNTs films synthesized at different conditions and the fitting curves. (**a**,**b**) 1223 K, (**c**,**d**) 1273 K, and (**e**,**f**) 1323 K.

The RBM peaks are Lorentz lines. As shown in Figure 4, we fitted the RBM peaks with the Formula (2):

(2) $$I\left(\omega \right)=I_{RBM}\frac{1}{1+{\left[2\left(\omega -\omega_{RBM}\right)/\Gamma_{RBM}\right]}^{2}},$$

where $I_{RBM}$ is the intensity of the RBM peak, $\omega_{RBM}$ is the RBM peak frequency at the maximum intensity, and $\Gamma_{RBM}$ is the FHWM of the RBM peak. The fitting data are shown in Table 2. $S_{RBM}$ is the area of the RBM peak. M-SWCNTs with the diameter of 1.28 nm and 1.24 nm were synthesized at 1273 K. m-SWCNTs with the diameter of 1.28 nm were synthesized at 1273 K. m-SWCNTs with the diameter of 1.24 nm and 1.14 nm were synthesized at 1323 K.

### 3.2. Carrier Flow

It can be considered that the pressure (P) of the carrier flow was constant. We obtained Formula (3) by ignoring the gas reaction loss:

(3) $$\frac{PV_{1}}{T_{1}}=\frac{PV_{2}}{T_{2}},$$

(4) $$V_{2}=\pi R_{g}^{2}{\bar{v}}_{a},$$

where $V_{1}$ is the flow rate at the gas inlet, $T_{1}$ is the temperature at the gas inlet, $V_{2}$ is the flow rate in the film-forming area, $T_{2}$ is the temperature in the film-forming area, ${\bar{v}}_{a}$ is the average velocity of the carrier flow, and $R_{g}$ is the inner radius of the glass tube.

According to Formulas (3) and (4),

(5) $${\bar{v}}_{a}=\frac{T_{2}V_{1}}{\pi R_{g}^{2}T_{1}}.$$

where $V_{1}=750\;\mathrm{mL}\cdot \min^{-1}$, $T_{1}=293\;K$, $T_{2}=1273\;K$, and $R_{g}=1.25\;\mathrm{cm}$,

(6) $${\bar{v}}_{a}=11.06\;\mathrm{cm}\cdot s^{-1}.$$

The Reynolds number (Re) of the experimental system is as follows:

(7) $$Re=\frac{{\bar{v}}_{a}d_{g}}{\nu },$$

where $d_{g}$ is the diameter of the glass tube, ν is the kinematic viscosity of the carrier flow, ${\bar{v}}_{a}=11.06\;\mathrm{cm}\cdot s^{-1}$, $d_{g}=2.5\;\mathrm{cm}$, and $\nu >1.403\times 10^{-5}\;m^{2}\cdot s^{-1}$ (the kinematic viscosity of Ar at 298 K),

(8) Re<197.1.

It is generally considered that the flow is laminar with Re<2000 and the velocity distribution of laminar flow in the tube is parabolic [20]. The velocity distribution of the carrier flow ($v_{a}$) is shown as follows:

(9) $$v_{a}=2{\bar{v}}_{a}\left(1-\frac{y^{2}+z^{2}}{R_{g}^{2}}\right).$$

### 3.3. Magnetic Field

**Figure 5:**
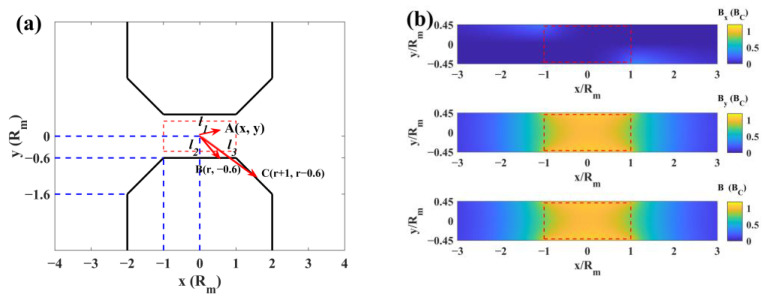
(**a**) The xy-plane of the electromagnet. (**b**) The simulation of the magnetic induction intensity distribution. The film-forming area is in the red rectangle.

We assumed that the magnetic charge density $\left(\sigma_{m}\right)$ on the surface was uniform. As shown in Figure 5a, the magnetic induction intensity (B) at point **A**, generated from the magnetic charge at points **B** and **C**, is as follows:

(10) $$dB=\sigma_{m}\left[\frac{\left(l_{1}-l_{2}\right)}{{\left|l_{1}-l_{2}\right|}^{3}}+\frac{\left(l_{1}-l_{3}\right)}{{\left|l_{1}-l_{3}\right|}^{3}}\right]dr,$$

Due to the rotational and mirror symmetry of the magnetic field, the magnetic induction intensity is as follows:

(11) $$B_{x}=\sigma_{m}\int_{0}^{2\pi }d\theta \int_{0}^{1}\left[\frac{\left(x-rcos\theta \right)r}{{\left[{\left(x-rcos\theta \right)}^{2}+r^{2}{sin}^{2}\theta +{\left(y+0.6\right)}^{2}\right]}^{\frac{3}{2}}}+\frac{\left(x-rcos\theta -1\right)\left(r+1\right)}{{\left[{\left(x-rcos\theta -1\right)}^{2}+{\left(r+1\right)}^{2}{sin}^{2}\theta +{\left(y-r+0.6\right)}^{2}\right]}^{\frac{3}{2}}}-\frac{\left(x+rcos\theta \right)r}{{\left[{\left(x+rcos\theta \right)}^{2}+r^{2}{sin}^{2}\theta +{\left(y-0.6\right)}^{2}\right]}^{\frac{3}{2}}}-\frac{\left(x+rcos\theta +1\right)\left(r+1\right)}{{\left[{\left(x+rcos\theta +1\right)}^{2}+{\left(r+1\right)}^{2}{sin}^{2}\theta +{\left(y+r-0.6\right)}^{2}\right]}^{\frac{3}{2}}}\right]dr,$$

(12) $$B_{y}=\sigma_{m}\int_{0}^{2\pi }d\theta \int_{0}^{1}\left[\frac{\left(y+0.6\right)r}{{\left[{\left(x-rcos\theta \right)}^{2}+r^{2}{sin}^{2}\theta +{\left(y+0.6\right)}^{2}\right]}^{\frac{3}{2}}}+\frac{\left(y-r+0.6\right)\left(r+1\right)}{{\left[{\left(x-rcos\theta -1\right)}^{2}+{\left(r+1\right)}^{2}{sin}^{2}\theta +{\left(y-r+0.6\right)}^{2}\right]}^{\frac{3}{2}}}-\frac{\left(y-0.6\right)r}{{\left[{\left(x+rcos\theta \right)}^{2}+r^{2}{sin}^{2}\theta +{\left(y-0.6\right)}^{2}\right]}^{\frac{3}{2}}}-\frac{\left(y+r-0.6\right)\left(r+1\right)}{{\left[{\left(x+rcos\theta +1\right)}^{2}+{\left(r+1\right)}^{2}{sin}^{2}\theta +{\left(y+r-0.6\right)}^{2}\right]}^{\frac{3}{2}}}\right]dr,$$

where $B_{x}$ is the induction intensity along the x-direction and $B_{y}$ is the induction intensity along the y-direction. According to Formulas (11) and (12), the magnetic field distribution is shown in Figure 5b.

## 4. Discussion

### 4.1. Dynamic Analysis of m-SWCNTs Flow

In the film-forming area, SWCNTs were mainly affected by the magnetic force, drag force, viscous force, etc. Since $\left|\chi_{m∥}\right|\gg \left|\chi_{s∥}\right|$ [3,4,5], the magnetic force on the m-SWCNTs was considered, while the effect of the magnetic field on s-SWCNTs was ignored. The motion behavior of m-SWCNTs flow was analyzed below.

The m-SWCNTs lined up along the direction of the magnetic field as a result of the magnetic field [21]. The magnetic dipole (m) of m-SWCNTs is as follows:

(13) $$m=\frac{m_{0}\chi_{∥}B}{\mu_{0}},$$

where $m_{0}$ is the mass of m-SWCNTs and $\mu_{0}$ is the vacuum magnetic permeability.

The magnetic force (F) on a small magnet is as follows:

(14) $$F=\nabla \left(m\cdot B\right),$$

**Figure 6:**
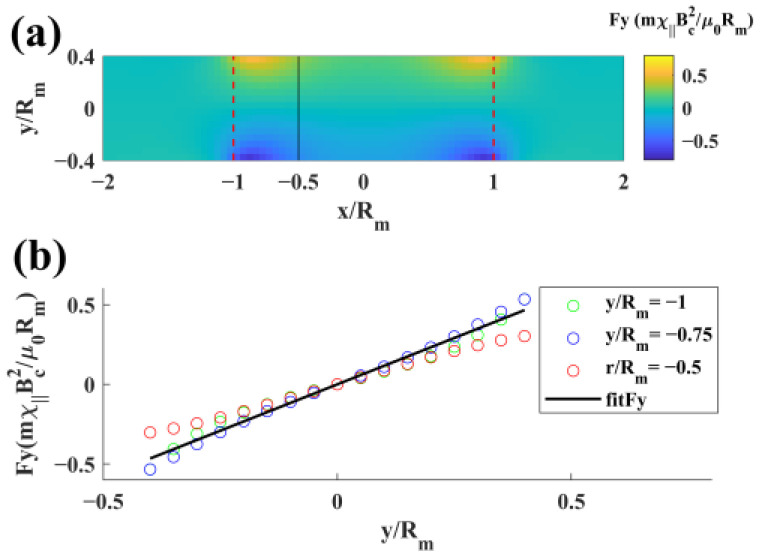
(**a**) Simulation distribution of the magnetic force along the y-direction. (**b**) Linear fitting of the magnetic force distribution in the region from *x* = −*R_m_* to *x* = −0.5*R_m_*.

As shown in Figure 5, the magnetic field area coincided with the film-forming area and the direction of the magnetic field was almost parallel to the y-direction. According to Formulas (13) and (14),

(15) $$F_{x}=0,$$

(16) $$F_{y}=2\frac{m_{0}\chi_{∥}B_{y}}{\mu_{0}}\frac{\partial B_{y}}{\partial x},$$

where $F_{x}$ is the magnetic force along the x-direction and $F_{y}$ is the magnetic force along the y-direction. The distribution of $F_{y}$ was obtained using Formulas (11), (12) and (16) and is shown in Figure 6a. As shown in Figure 6b, $F_{y}$ was simplified to Formula (17):

(17) $$F_{y}=2.328\frac{m_{0}\chi_{∥}B_{c}^{2}}{\mu_{0}R_{m}^{2}}y,$$

where $R_{m}$ is the radius of the electromagnet.

Due to the viscous force and its rod-like structure, we assumed that the velocity of the m-SWCNTs in the x-direction is equal to $v_{a}$ and ignored the viscous force in the y-direction. The motion equations of m-SWCNTs are as follows:

(18) $$\frac{dx}{dt}=v_{a},$$

(19) $$\frac{d^{2}y}{dt^{2}}=\frac{F_{y}}{m_{0}},$$

(20) $${\left.x\right|}_{t=0}=-R_{m},$$

(21) $${\left.\frac{dy}{dt}\right|}_{t=0}=0,$$

(22) $${\left.y\right|}_{t=0}=y_{0},$$

(23) z=0.

According to Formulas (9) and (17)–(23), the trajectory equation of m-SWCNTs is as follows:

(24) $$x=\frac{1.31\mu_{0}^{\frac{1}{2}}R_{m}{\bar{v}}_{g}}{\chi_{\|}^{\frac{1}{2}}B_{c}}\left[\left(1-\frac{y_{0}^{2}}{2R_{g}^{2}}\right)\ln\left(\frac{y}{y_{0}}+\sqrt{\frac{y^{2}}{y_{0}^{2}}-1}\right)-\frac{yy_{0}}{2R_{g}^{2}}\sqrt{\frac{y^{2}}{y_{0}^{2}}-1}\right]-R_{m},$$

### 4.2. Calculation of the Axial Magnetic Susceptibility

Assuming that m-SWCNTs were not produced in the film-forming area, we obtained Formulas (25) and (26):

(25) $$Q_{0}\left|dy_{0}\right|=Q_{1}\left|dx_{1}\right|,\;Q_{0}=\rho v_{0},$$

(26) $$x_{1}=\frac{1.31\mu_{0}^{\frac{1}{2}}R_{m}{\bar{v}}_{g}}{\chi_{\|}^{\frac{1}{2}}B_{c}}\left[\left(1-\frac{y_{0}^{2}}{2R_{g}^{2}}\right)\ln\left(\frac{R_{g}}{y_{0}}+\sqrt{\frac{R_{g}^{2}}{y_{0}^{2}}-1}\right)-\frac{y_{0}}{2R_{g}}\sqrt{\frac{R_{g}^{2}}{y_{0}^{2}}-1}\right]-R_{m},$$

where $Q_{0}$ is the flux density of m-SWCNTs flow in the x-direction at $x=-R_{m}\;\mathrm{and}\;y=y_{0}$, $Q_{1}$ is the flux density of m-SWCNTs flow in the y-direction at $x=x_{1}\;\mathrm{and}\;y=R_{g}$, ρ is the density of m-SWCNTs flow before entering the film-forming area, and $v_{0}$ is the velocity of m-SWCNTs flow at $x=-R_{m}\;\mathrm{and}\;y=y_{0}$. Since the SWCNTs could not be synthesized near the tube wall [22], ρ approached zero as $y_{0}$ approached $R_{g}$.

According to Formulas (25) and (26),

(27) $$B_{c}=k_{1}\left[\left(1-\frac{y_{0}^{2}}{2R_{g}^{2}}\right)\ln\left(\frac{R_{g}}{y_{0}}+\sqrt{\frac{R_{g}^{2}}{y_{0}^{2}}-1}\right)-\frac{y_{0}}{2R_{g}}\sqrt{\frac{R_{g}^{2}}{y_{0}^{2}}-1}\right]\;,\;k_{1}=\frac{1.31\mu_{0}^{\frac{1}{2}}R_{m}{\bar{v}}_{g}}{\chi_{\|}^{\frac{1}{2}}\left(x_{1}+R_{m}\right)},$$

(28) $$Q_{1}=k_{2}\left(1-\frac{y_{0}^{2}}{R_{g}^{2}}\right)\left[\frac{\ln\left(\frac{R_{g}}{y_{0}}+\sqrt{\frac{R_{g}^{2}}{y_{0}^{2}}-1}\right)}{\frac{y_{0}}{R_{g}}\ln\left(\frac{R_{g}}{y_{0}}+\sqrt{\frac{R_{g}^{2}}{y_{0}^{2}}-1}\right)+\sqrt{\frac{R_{g}^{2}}{y_{0}^{2}}-1}}-\frac{y_{0}}{2R_{g}}\right]\;,\;k_{2}=\frac{2\rho {\bar{v}}_{g}R_{m}}{\left(x_{1}+R_{m}\right)},$$

**Figure 7:**
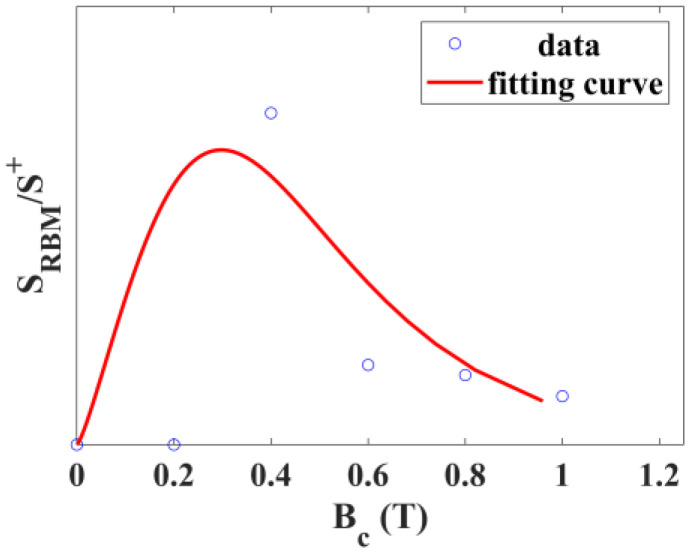
Fitting curve of $S_{RBM}/S^{+}$ of SWCNTs films synthesized at 1273 K, varying with the central magnetic induction intensity.

As shown in Figure 3b and Figure 7, both the $S_{RBM}/S^{+}$ and $S^{-}/S^{+}$ of SWCNTs films synthesized at 1273 K have a peak at 0.4 T, indicating that the quantity of a type of m-SWCNTs ($d_{t}=1.28\;\mathrm{nm}$) synthesized at 1273 K reached its maximum at 0.4 T. In contrast, the $S^{-}/S^{+}$ of SWCNTs films synthesized at 1223 K and 1323 K has no peak at 0.4 T, consistent with the finding that the Raman spectra (Figure 4) of these samples have no RBM peak similar to the m-SWCNTs ($d_{t}=1.28\;\mathrm{nm}$). The quantity of this type of m-SWCNTs is negligible at 1223 K and cannot be synthesized at 1323 K. It can be concluded that $S_{RBM}/S^{+}$ characterizes the quantity of a type of m-SWCNTs ($d_{t}=1.28\;\mathrm{nm}$) at the sampling position, and $S^{-}/S^{+}$ characterizes the quantity of m-SWCNTs at the sampling position. $S_{RBM}/S^{+}$ is proportional to $Q_{1}$:

(29) $$S_{RBM}/S^{+}=k_{3}Q_{1},$$

where $k_{3}$ is a constant. With the weak magnetic field, m-SWCNTs in the samples mainly came from the sites near the tube wall.$\;S_{RBM}/S^{+}$ is zero at 0 T and 0.2 T, indicating that this kind of m-SWCNTs could not be synthesized near the tube wall. To simplify the calculation, we took ρ as a constant and fitted $S_{RBM}/S^{+}$ with Formulas (27)–(29), except for 0.2 T. The fitting curve is shown in Figure 7. The fitting result was as follows:

(30) $$k_{1}=0.2009\;T.$$

where $\mu_{0}=4\pi \times 10^{-7}N\cdot A^{-1}\cdot m^{-1}$, $R_{m}=3\;\mathrm{cm}$, $x_{1}=-1.5\;\mathrm{cm}$, and ${\bar{v}}_{a}=11.06\;\mathrm{cm}\cdot s^{-1}$,

(31) $$\chi_{\|}=2.61\times 10^{-6}\;m^{3}\cdot {\mathrm{kg}}^{-1}.$$

The SWCNTs films synthesized at 1223 K and 1.0 T, 1323 K and 0.8 T, and 1323 K and 1.0 T had fewer defects. This phenomenon could be because m-SWCNTs with defects had high axial magnetic susceptibilities, were subject to high magnetic forces, and were deposited on the substrates before the sampling location.

This is the first time that the $\chi_{\|}$ of m-SWCNTs has been measured. Previous studies on magnetic susceptibility and anisotropy are shown in Table 3. The χ of m-SWCNTs, without distinguishing $\chi_{∥}$ and $\chi_{┴}$, is interpreted as follows:

(32) $$\chi =\alpha \chi_{∥}+\left(1-\alpha \right)\chi_{┴},0<\alpha <1.$$

where α is a variable related to the arrangement of m-SWCNTs. It can be concluded that

(33) $$\chi <\chi_{∥}<∆\chi .$$

According to Table 3, the magnitude of the $\chi_{∥}$ of purified m-SWCNTs is not more than $10^{-8}\;m^{3}\cdot {kg}^{-1}$ at 300 K. The $\chi_{∥}$ of m-SWCNTs ($d_{t}=1.28\;\mathrm{nm}$) with defects at 1273 K is at least two orders of magnitude greater than that of purified m-SWCNTs at 300 K. Combining the experimental results with the Curie–Weiss law, it can be concluded that the defects increase the Curie temperature and Curie constant of m-SWCNTs. The spin-polarized density functional theory predicts that m-SWCNTs with vacancies have local magnetic moments around the vacancies and exhibit ferro- or ferrimagnetism [7]. The experimental results are consistent with the spin-polarized density functional theory.

## 5. Conclusions

In a magnetic field-assisted FC-CVD, some SWCNTs films were synthesized under different magnetic fields and temperatures. The content of m-SWCNTs in the films varied with the magnetic field, and a model was built to describe this change by analyzing the motion behavior of m-SWCNTs flow in the field-assisted FC-CVD. The axial magnetic susceptibility of m-SWCNTs was obtained as a parameter in the model. The axial magnetic susceptibility of m-SWCNTs ($d_{t}=1.28\;\mathrm{nm}$) with defects was $2.61\times 10^{-6}\;m^{3}\cdot {\mathrm{kg}}^{-1}$ at 1273 K. This is the first time that the axial magnetic susceptibility of m-SWCNTs has been obtained, and the result at 1273 K is at least two orders of magnitude greater than purified m-SWCNTs at 300 K. This difference is caused by the defects and conforms to the spin-polarized density functional theory, which predicts that m-SWCNTs with vacancies have local magnetic moments around the vacancies and exhibit ferro- or ferrimagnetism [7]. This study introduces a novel approach to investigating the magnetic properties of SWCNTs and provides the basis for the precise preparation of SWCNTs materials.

## Figures and Tables

**Table 1:** Fitting data of G peaks.

*T* (K)	$B_{c}\;(T)$	$I^{+}$	$\omega^{+}\left({\mathrm{cm}}^{-1}\right)$	$\Gamma^{+}\left({\mathrm{cm}}^{-1}\right)$	$I^{-}$	$\omega^{-}\left({\mathrm{cm}}^{-1}\right)$	$\Gamma^{-}\left({\mathrm{cm}}^{-1}\right)$	$q^{-1}$	$S^{+}$	$S^{-}$
1223	0	0.4384	1612	25.50	0.5444	1587	32.74	−0.2081	17.56	26.7
	0.2	0.3455	1616	22.48	0.6073	1590	37.16	−0.1316	12.20	34.83
	0.4	0.4223	1610	28.28	0.4967	1586	31.50	−0.2168	18.76	23.42
	0.6	0.3707	1613	25.26	0.5962	1587	34.36	−0.1726	14.71	31.22
	0.8	0.2037	1618	19.46	0.6953	1593	38.66	−0.1132	6.228	41.68
	1.0	0.7784	1595	28.10	0.2432	1573	41.14	−0.1536	34.36	15.35
1273	0	0.2923	1617	23.44	0.5824	1598	39.22	−0.1337	10.76	35.24
	0.2	0.3489	1616	24.86	0.5358	1594	39.52	−0.1574	13.62	32.44
	0.4	0.2461	1617	21.82	0.6965	1591	41.76	−0.1216	8.44	45.01
	0.6	0.3214	1614	24.16	0.6501	1586	35.04	−0.1311	12.20	35.17
	0.8	0.2212	1616	22.00	0.7290	1592	43.60	−0.1249	7.64	49.15
	1.0	0.3275	1617	26.20	0.6099	1593	40.50	−0.144	13.48	38.00
1323	0	0.3128	1613	26.24	0.6753	1588	37.74	−0.1371	12.89	39.28
	0.2	0.3303	1613	28.48	0.5894	1585	33.70	−0.1321	14.77	30.66
	0.4	0.3177	1614	26.44	0.6397	1589	37.64	−0.1495	13.19	36.98
	0.6	0.3251	1610	14.00	0.5491	1584	17.32	−0.1586	14.30	29.13
	0.8	0.8965	1592	16.78	0.1399	1567	13.89	−0.3866	47.26	5.192
	1.0	0.8340	1595	16.30	0.2513	1565	25.53	−0.1833	42.71	19.48

**Table 2:** Fitting data of RBM peaks.

*T* (K)	$B_{c}\;(T)$	$I_{RBM}$	$\omega_{RBM}\;({\mathrm{cm}}^{-1})$	$\Gamma_{RBM}\;({\mathrm{cm}}^{-1})$	$S_{RBM}$
1223	0				0
	0.2				0
	0.4				0
	0.6	0.05434	193.8	17.6	1.50
	0.8				0
	1.0	0.03577	199.4	25.8	1.45
1273	0				0
	0.2				0
	0.4	0.1149	191.3	17.7	3.193
	0.6	0.05412	194.2	13.1	1.110
	0.8	0.04094	194.1	22.1	1.421
	1.0	0.05828	196.4	8.2	0.746
1323	0				0
	0.2				0
	0.4				0
	0.6				0
	0.8	0.04209	200.8	28.52	1.886
		0.04164	218.6	12.94	0.846
	1.0	0.1005	199.8	14.83	2.341
		0.05231	217.2	14.84	1.219

**Table 3:** The magnetic susceptibility (anisotropy) of SWCNTs.

	T(K)	$d_{t}\left(\mathrm{nm}\right)$	$∆\chi \left(m^{3}\cdot {kg}^{-1}\right)$	$\chi \left(m^{3}\cdot {kg}^{-1}\right)$	$\chi_{\|}\left(m^{3}\cdot {kg}^{-1}\right)$	
s-SWCNTs		1	$1.47\times 10^{-8}$			8
(6, 4)-SWCNTs		0.69	$1.51\times 10^{-8}$			9
(6, 5)-SWCNTs		0.76	$1.27\times 10^{-8}$			
(8, 3)-SWCNTs		0.78	$1.35\times 10^{-8}$			
(7, 5)-SWCNTs		0.82	$1.57\times 10^{-8}$			
(6, 4)-SWCNTs	300	0.69	$1.30\times 10^{-8}$			10
(6, 5)-SWCNTs	300	0.76	$1.06\times 10^{-8}$			
(8, 3)-SWCNTs	300	0.78	$2.23\times 10^{-8}$			
(7, 5)-SWCNTs	300	0.83	$1.74\times 10^{-8}$			
(6, 6)-SWCNTs	300	0.83	$3.80\times 10^{-8}$			
(5, 5)-SWCNTs	300	0.69	$3.51\times 10^{-8}$			
(7, 4)-SWCNTs	300	0.77	$2.56\times 10^{-8}$			
Purified SWCNTs	300	1.0		$-1.39\times 10^{-8}$		15
Purified SWCNTs	300	1.4		$-2.04\times 10^{-8}$		
Purified SWCNTs	300	1.9		$-2.74\times 10^{-8}$		
Purified SWCNTs	300	2.65		$-3.90\times 10^{-8}$		
Purified SWCNTs and s-SWCNT-rich	300	1.4		$-1.86\times 10^{-8}$		
Purified SWCNTs and m-SWCNT-rich	300	1.4		$-1.27\times 10^{-8}$		
m-SWCNTs with defects	1273	1.3			$2.61\times 10^{-6}$	This work

**Figure A1:**
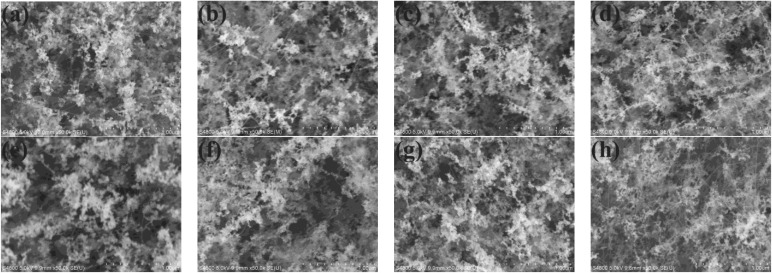
SEM morphologies of SWCNTs films synthesized under different conditions: (**a**) 0 T and 1223 K, (**b**) 0 T and 1323 K, and (**c**–**h**) 0 T, 0.2 T, 0.4 T, 0.6 T, 0.8 T, and 1.0 T and 1273 K.

## Data Availability

The raw data supporting the conclusions of this article will be made available by the authors on request.

## Appendix A

As shown in Figure A1, SWCNTs films were synthesized under different conditions. SWCNTs formed networks at the mesoscopic scale and were surrounded by particles. With the adjustment of preparation conditions, the number of particles was almost constant, except at 1.0 T and 1273 K. This difference was caused by the different quality of SWCNTs films.
